# PTEN loss represses glioblastoma tumor initiating cell differentiation via inactivation of Lgl1

**DOI:** 10.18632/oncotarget.1164

**Published:** 2013-07-21

**Authors:** Alexander Gont, Jennifer E L Hanson, Sylvie J Lavictoire, Doris A E Parolin, Manijeh Daneshmand, Ian J Restall, Mathieu Soucie, Garth Nicholas, John Woulfe, Amin Kassam, Vasco F Da Silva, Ian AJ Lorimer

**Affiliations:** ^1^ Centre for Cancer Therapeutics, Ottawa Hospital Research Institute, 501 Smyth Road, Ottawa, K1H 8L6, Canada; ^2^ Department of Biochemistry, Microbiology and Immunology, University of Ottawa, Ottawa, Ontario, Canada; ^3^ Faculty of Science, University of Ottawa, Ottawa, Ontario, Canada; ^4^ Department of Pathology and Laboratory Medicine, University of Ottawa, Ottawa, Ontario, Canada; ^5^ Department of Surgery, University of Ottawa, Ottawa, Ontario, Canada; ^6^ Department of Medicine, University of Ottawa, Ottawa, Ontario, Canada

**Keywords:** glioblastoma, glioma, PTEN, PKCι, Lgl, tumor initiating cell

## Abstract

Glioblastoma multiforme is an aggressive and incurable type of brain tumor. A subset of undifferentiated glioblastoma cells, known as glioblastoma tumor initiating cells (GTICs), has an essential role in the malignancy of this disease and also appears to mediate resistance to radiation therapy and chemotherapy. GTICs retain the ability to differentiate into cells with reduced malignant potential, but the signaling pathways controlling differentiation are not fully understood at this time. PTEN loss is a very common in glioblastoma multiforme and leads to aberrant activation of the phosphoinositide 3-kinase pathway. Increased signalling through this pathway leads to activation of multiple protein kinases, including atypical protein kinase C. In Drosophila, active atypical protein kinase C has been shown to promote the self-renewal of neuroblasts, inhibiting their differentiation along a neuronal lineage. This effect is mediated by atypical protein kinase c-mediated phosphorylation and inactivation of Lgl, a protein that was first characterized as a tumour suppressor in Drosophila. The effects of the atypical protein kinase C/Lgl pathway on the differentiation status of GTICs, and its potential link to PTEN loss, have not been assessed previously. Here we show that PTEN loss leads to the phosphorylation and inactivation of Lgl by atypical protein kinase C in glioblastoma cells. Re-expression of PTEN in GTICs promoted their differentiation along a neuronal lineage. This effect was also seen when atypical protein kinase C was knocked down using RNA interference, and when a non-phosphorylatable, constitutively active form of Lgl was expressed in GTICs. Thus PTEN loss, acting via atypical protein kinase C activation and Lgl inactivation, helps to maintain GTICs in an undifferentiated state.

## INTRODUCTION

Glioblastoma multiforme is an aggressive type of adult brain tumor. Surgery is not curative, as this disease invariably exhibits extensive intracerebral dissemination at the time of diagnosis. Patients are also commonly treated with radiation and the alkylating agent temozolomide; this prolongs survival but is also not curative [1]. A characteristic histological feature of glioblastoma is that glioblastoma cells show considerable phenotypic heterogeneity. One factor contributing to this heterogeneity is that glioblastoma cells exist in different differentiation states [2]. A subset of glioblastoma cells are relatively undifferentiated; these are variously referred to as glioblastoma stem cells, glioblastoma stem-like cells or glioblastoma tumor-initiating cells [3,4]. The latter term is based on one of their defining properties, which is the ability to regenerate a tumor resembling the patient's original tumor when implanted in the brain of an immunocompromised mouse [3]. These cells share some properties with normal adult neural stem cells, including the expression of stem cell-associated genes and the ability to differentiate along multiple lineages [5]. Current data suggests that these undifferentiated cells are resistant to radiation therapy and temozolomide and therefore play a key role in glioblastoma recurrence [6,7].

The genetics of glioblastoma are now understood in some detail. Loss of PTEN is a very frequent event in glioblastoma, with hemizygous or homozygous deletions occurring in over 90% of primary glioblastomas [8]. PTEN catalyzes the inactivation of the second messenger phosphatidylinositol 3,4,5 trisphosphate. This second messenger is produced by the enzyme phosphoinositide 3' kinase (PI 3-kinase) after activation by tyrosine kinase receptors. Loss of PTEN therefore results in increased signaling through the PI 3-kinase pathway. Other glioblastoma mutations, including mutations in the *EGFR*, *PIK3CA* and *PIK3R1* genes, also activate this pathway [8,9]. While much attention has focused on the role of Akt/PKB as a downstream mediator in the PI 3-kinase pathway, PI 3-kinase signaling results in the activation of multiple other downstream kinases [10]. This includes atypical protein kinase C (PKC) family members [11]. There are two atypical PKCs in humans, PKCζ and PKCι. Of these, PKCι is the most ubiquitously expressed in tissues and overexpressed PKCι has been shown to have the properties of an oncogene in several different tumor types [12]. In studies using human glioblastoma cell lines, PKCι has been shown to have a role in both proliferation and invasion [13,14,15]. Relatively little is known about the kinase substrates that mediate these effects. One of the more well-characterized substrates of the atypical PKCs is a protein known as Lgl.

Lethal Giant larvae (Lgl) was first identified as an allele in *Drosophila* that, when mutated, gave rise to a neoplastic phenotype characterized by overgrowth of imaginal epithelia and brain tissue [16]. In *Drosophila* brain tissue, this overgrowth is the result of neuroblasts preferentially undergoing self-renewal rather than differentiating into neurons [17]. Mammals have two genes with homology to *Drosophila* Lgl: *LGL1*, which in mice is broadly expressed with the highest expression in brain; and *LGL2*, which shows a more restricted expression pattern [18]. Knockout of Lgl1 in mice causes a brain dysplasia phenotype [18]. At the cellular level, Lgl proteins have multiple functions related to cell polarity, including asymmetric cell division [19]. Additional cell polarity functions include the maintenance of apical/basolateral cell polarity (in epithelial tissue); polarized exocytosis; and cell motility [20,21]. Lgl proteins bind non-muscle myosin II and associate with the inner leaflet of the plasma membrane. They also bind the scaffolding protein Par6. Atypical PKCs also bind to Par6 and in this complex they are able to phosphorylate and inactivate Lgl. Inactivated Lgl no longer associates with the plasma membrane or with non-muscle myosin II [22].

Given its original characterization as a tumor suppressor in *Drosophila*, a possible role for Lgl as a tumor suppressor in human cancers has also been investigated. Human Lgl1 can rescue *Drosophila* Lgl mutants, showing conservation of function [23]. Human Lgl1 mRNA and protein are reduced in multiple cancer types including colorectal cancer and melanoma [23,24,25]. This reduced expression is not due to either Lgl1 gene mutations or promoter methylation, but instead is due to transcriptional repression [26]. Although Lgl1 shows strong expression in brain and is known to control brain development in both *Drosophila* and mammals, there has been no detailed investigation of the role of Lgl1 in glioblastoma to date. Here we show that in glioblastoma, PTEN loss results in the inactivation of Lgl1 by phosphorylation. This inactivation of Lgl1 has a key function in the maintenance of undifferentiated glioblastoma tumor-initiating cell populations.

## RESULTS

### Constitutive phosphorylation of Lgl1 in glioblastoma cells

**Figure 1:**
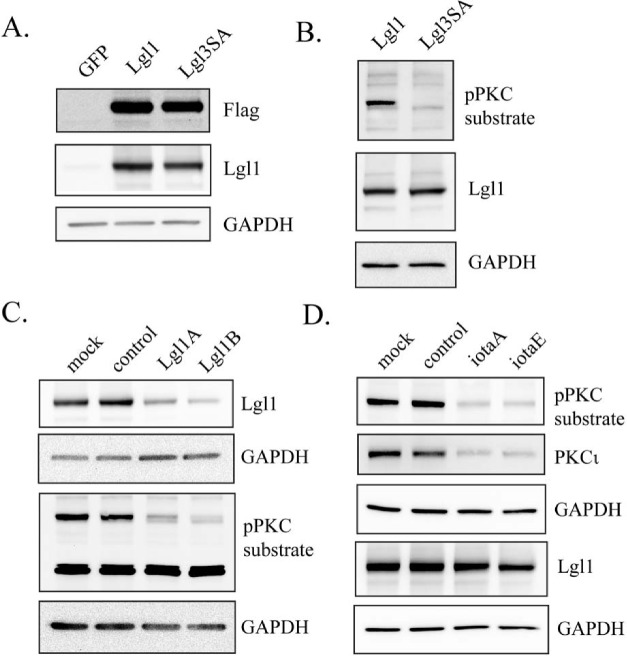
Lgl1 is constitutively phosphorylated by PKCι in PTEN-null U87MG cells A. U87MG cells were transduced with lentiviral vectors expressing GFP (as a control), wild-type Lgl1, or a mutant non-phosphorylatable Lgl1 (Lgl3SA). Expression of Lgl1 was assessed by Western blotting of total cell lysates using antibodies to Flag epitope, Lgl1 and GAPDH (as a loading control); B. U87MG cells were transduced with lentiviral vectors expressing either wild-type Lgl1 or Lgl3SA. Total cell lysates were analyzed by Western blotting using antibodies to phospho-PKC substrate, Lgl1 and GAPDH; C. U87MG cell transduced with lentiviral vector expressing wild-type Lgl1 were mock transfected, transfected with control RNA duplex, or transfected with two different RNA duplexes targeting Lgl1 (LglA and LglB). 48 h after transfection, total cell lysates were collected and analyzed on separate Western blots with antibodies to Lgl1and phospho-PKC substrate (GAPDH loading controls are shown for each blot). D. U87MG cell transduced with lentiviral vector expressing wild-type Lgl1 were mock transfected, transfected with control RNA duplex, or transfected with two different RNA duplexes targeting PKCι (iotaA and iotaE). 48 h after transfection, total cell lysates were collected and analyzed by Western blotting for phospho-PKC substrate, PKCι and Lgl1.

A lentiviral vector for constitutive expression of Lgl1 was constructed and used to express Lgl1 in U87MG human glioblastoma cells. In addition a second lentiviral vector was made to express a non-phosphorylatable, constitutively active Lgl1 (designated Lgl3SA), in which the three major Lgl1 phosphorylation sites identified by Yamanaka *et* *al*. were mutated to alanine [27]. Transduced Lgl1 and Lgl3SA were expressed at similar levels in U87MG glioblastoma cells (Figure 1A). U87MG cells express low levels of endogenous Lgl1, visible as a faint band in Figure 1A in the blot probed with Lgl1 antibody. To detect phosphorylated transduced Lgl1, a phospho-(Ser) PKC substrate antibody was used; in total cell extracts this labeled a prominent band of the expected size in cells transduced with Lgl1, but not in cells transduced with Lgl3SA (Figure 1B). Knockdown of Lgl1 with two different Lgl1 RNA duplexes also decreased the intensity of the band detected with the phospho-(Ser) PKC substrate antibody, confirming its identity (Figure 1C). Knockdown of PKCι (the sole atypical PKC isoform expressed in U87MG cells [14]) also reduced the intensity of this band, confirming that PKCι is responsible for Lgl1 phosphorylation in these cells (Figure 1D).

### Effects of PTEN on Lgl1 activation

**Figure 2:**
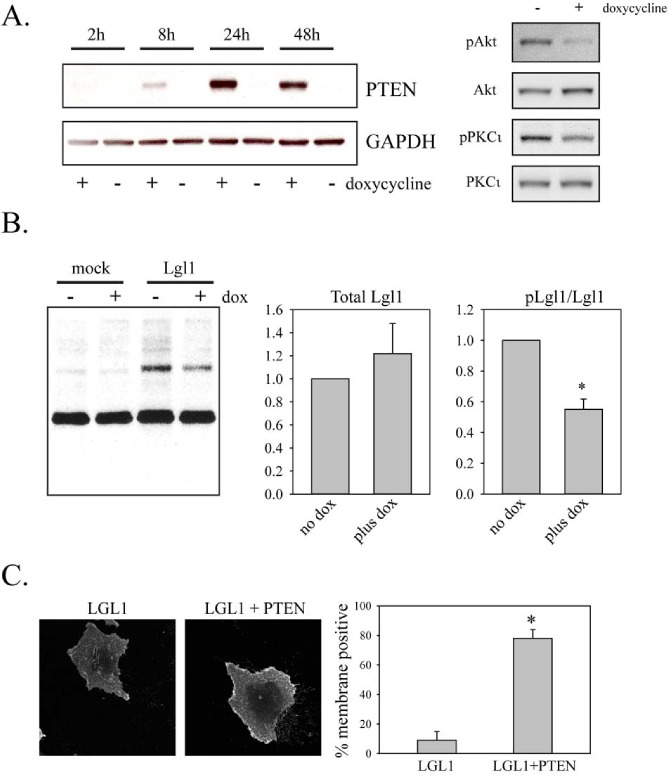
Restoration of PTEN expression reduces Lgl1 phosphorylation and promotes its membrane association A. U87MG cells were transduced with lentiviral vectors expressing Tet activator and Tet-inducible PTEN. Cells were treated with or without 100 ng/ml doxycycline for the indicated period of time. Total cell lysates were then analyzed by Western blotting for PTEN expression (left panels). Total cell lysates from cell treated for 48 h with 100 ng/ml doxycycline were also analyzed by Western blotting for total and phosphorylated Akt and PKCι (right panels). B. U87MG cells with doxycycline-inducible PTEN were treated for two days with doxycycline and then either mock-transduced or transduced with lentiviral vector constitutively expressing wild-type Lgl1. Total cell lysates were collected two days after transduction and analyzed by Western blotting with phospho-PKC substrate antibody (shown), Lgl1 antibody and GAPDH antibody (not shown). Data from three independent experiments were analyzed for levels of total and phosphorylated Lgl1 (bar graphs at left). Data were first normalized to GAPDH levels and are shown normalized to the no doxycycline condition in the bar graphs. Data are shown as the mean ± SD. * indicates a p value less than 0.05. C. U87MG cells with doxycycline-inducible PTEN were treated for two days with doxycycline and then transduced with lentiviral vector expressing wild-type Lgl1. Two days after transduction, cells were fixed and analyzed by immunofluorescence with anti-Flag antibody. Examples of immunofluorescence staining are shown in the left two panels. The right panel shows the analysis for membrane localization from 15 randomly selected images per experiment assessed by an observer (IR) blinded to the treatment conditions. Data are the mean SD from three separate experiments. * indicates a p value less than 0.05.

To assess the effects of PTEN on Lgl1 phosphorylation, a doxycycline-inducible expression system for PTEN was generated in U87MG cells, which do not express PTEN due to a mutation [28]. This gave rapid, inducible expression of PTEN that was active, as assessed by its ability to decrease phosphorylation of Akt and PKCι (Figure 2A). These cells were transduced with Lgl1 cDNA and Lgl1 phosphorylation was assessed with phospho-(Ser) PKC substrate antibody as above. Induction of PTEN decreased levels of phosphorylated Lgl1 without significantly affecting the total levels of Lgl1 protein (Figure 2B). Lgl1 in its active form is membrane-localized and inactivation by phosphorylation causes its translocation to the cytosol. In U87MG cells, transduced wild-type Lgl1 was localized in the cytoplasm. Induction of PTEN altered the subcellular localization of wild-type Lgl1, such that it was predominantly membrane-associated, consistent with its restoration to an active form (Figure 2C).

### Membrane association of a non-phosphorylatable Lgl1 mutant in glioblastoma cells

**Figure 3:**
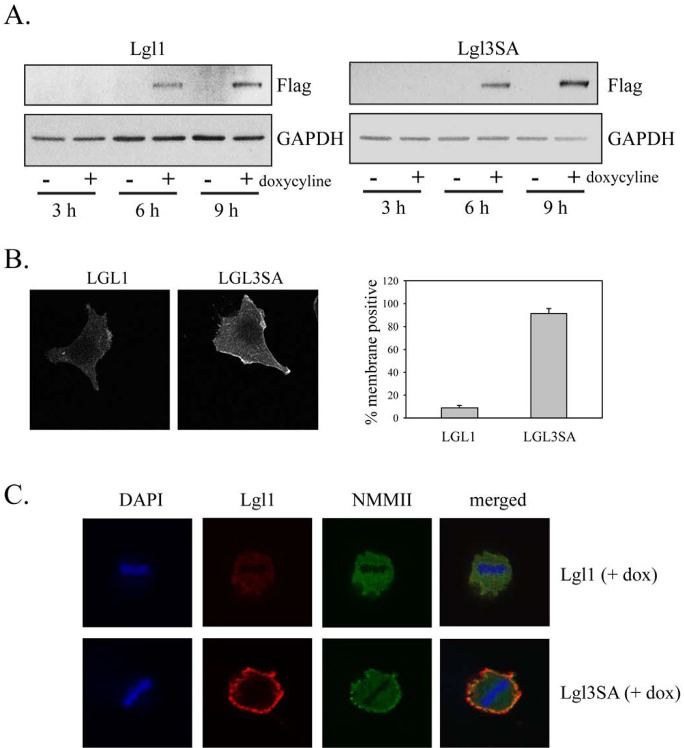
Non-phosphorylatable Lgl1 constitutively associates with the cell membrane in U87MG cells U87MG cells were transduced with lentiviral vectors expressing tet activator and tet-inducible Lgl or Lgl3SA. Cells were treated with 100 ng/ml doxycycline for the indicated periods of time and then analyzed by Western blotting with anti-Flag antibody. B. U87MG cells with inducible Lgl1 or Lgl3SA expression were treated with 100 ng/ml doxycycline for 4 days and then analyzed by immunofluorescence microscopy using anti-Flag antibody. Membrane localization was quantitated as describe in Figure 2C. C. U87MG cells with inducible Lgl1 or Lgl3SA expression were treated with 100 ng/ml doxycycline for 4 days and then analyzed by confocal immunofluorescence microscopy with antibodies to Flag epitope (red) and non-muscle myosin IIa (green). Cells were counterstained with DAPI. Examples of confocal images of mitotic cells expressing Lgl1 and Lgl3SA are shown.

A doxycycline-inducible expression system to express either wild-type Lgl or Lgl3SA was also made in U87MG cells. As with the constitutive expression system, both proteins were expressed at similar levels and were detectable within 6 h after induction of expression with doxycycline (Figure 3A). Induced Lgl and Lgl3SA showed different subcellular localizations in glioblastoma cells, with Lgl being predominantly cytoplasmic and Lgl3SA showing significant membrane association (Figure 3B). This membrane localization was particularly pronounced in mitotic cells; double staining for both Lgl and non-muscle myosin II (a known Lgl binding partner) in mitotic cells showed that Lgl3SA, but not Lgl, colocalized with non-muscle myosin II.

### Characterization of glioblastoma tumor initiating cells (GTICs)

**Figure 4:**
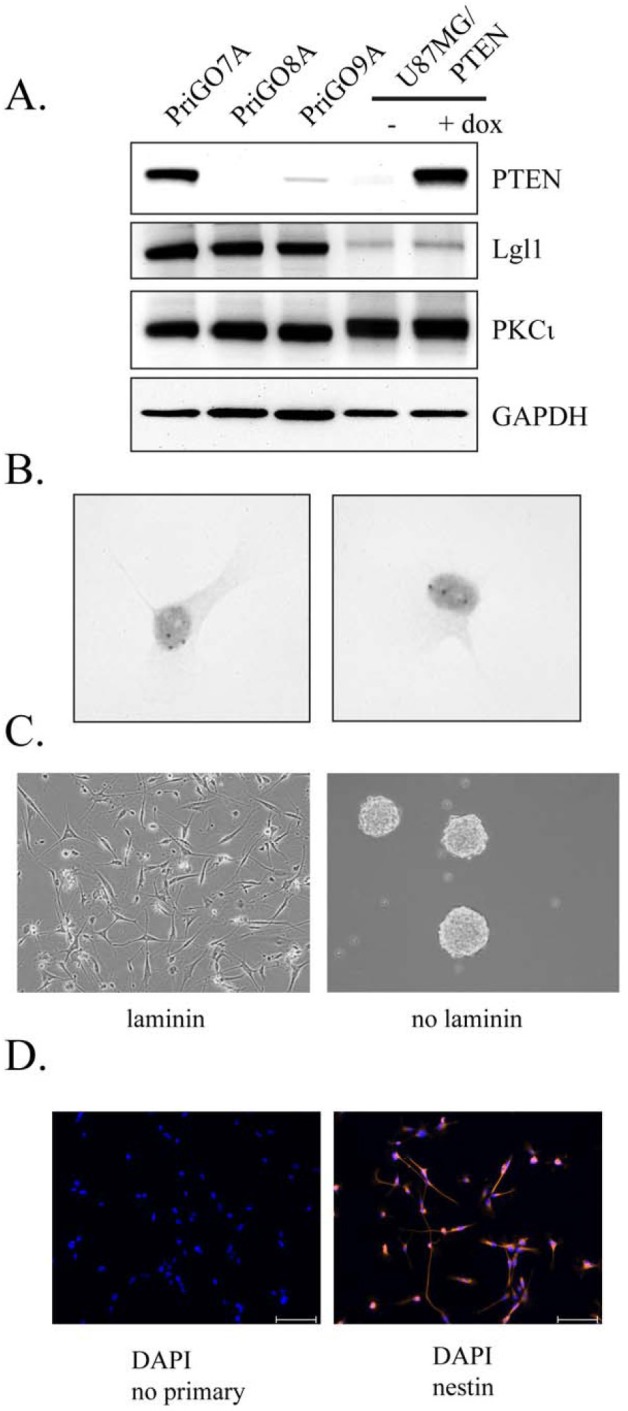
Characterization of PriGO8A cells A. Western blot analysis of PTEN, Lgl1 and PKCι expression in GTIC cultures from three patients. Total cell extracts from U87MG cells with doxycycline-inducible PTEN were also run for comparison. B. Chromogenic in situ hybridization was performed on PriGO8A cells using an EGFR probe. Examples of two nuclei are shown. The three dark spots in the nuclei indicate a gain for this region of chromosome 7. C. Left panel, phase contrast microscopy of PriGO8A cells grown in the presence of laminin; right panel, phase contrast microscopy of PriGO8A cells grown in the absence of laminin, showing neurosphere formation. D. Immunofluorescence microscopy for nestin in PriGO8A cells growing on laminin. Cells were counterstained with DAPI. A control in which the primary antibody was omitted during staining is also shown.

**Figure 5:**
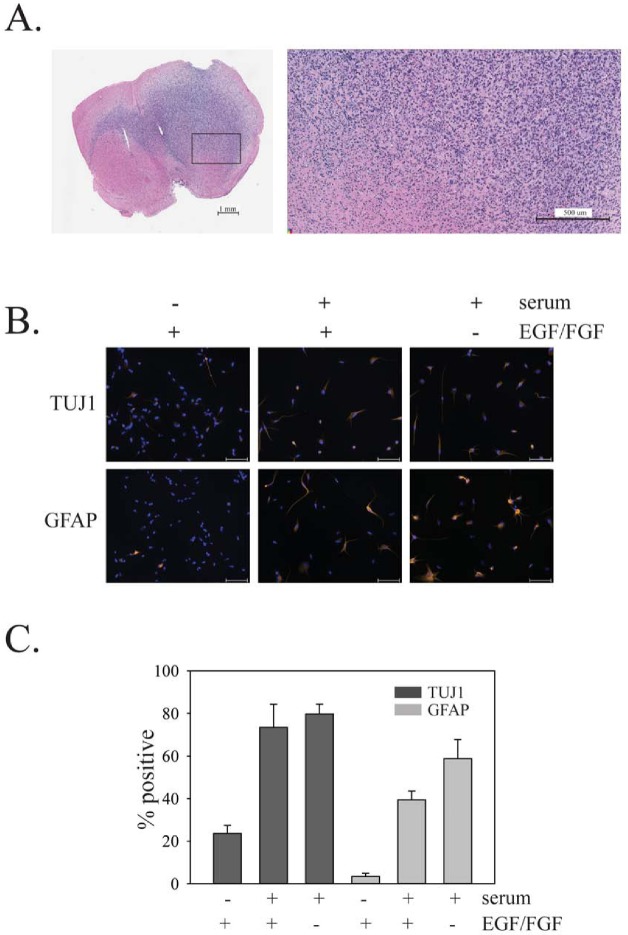
In vivo growth of PriGO8A cells and differentiation of PriGO8A cells in response to serum and/or growth factor withdrawal A. Growth of PriGO8A cells three months after intrastriatal injection in the right hemisphere. Hematoxylin and eosin stained whole brain sections (left panel) show extensive growth and invasion into the left hemisphere across the corpus callosum. The close-up (right panel) shows a border area between cancer cells and normal brain. B.PriGO8A cells were grown in: regular media; media supplemented with 10% fetal calf serum; media supplemented with 10% fetal calf serum without EGF and FGF growth factors. Seven days later cells were fixed and immunofluorescence microscopy was performed for either TUJ1 or GFAP. C. Quantitation of the results in B. Data are shown as the mean ±SE.

GTICs were isolated from patients undergoing surgery for glioblastoma at The Ottawa Hospital. Cells were isolated following the procedure described by Pollard *et al*., in which GTICs are plated directly onto laminin-coated plates [5]. Cells were cultured in 5% O_2_, which significantly enhanced their growth rate compared to growth in atmospheric oxygen levels (20%). Enhanced growth in 5% O_2_ has been observed previously for neural stem cells [29] and may be due in part to reduced senescence [30]. This level is also more physiologically appropriate than atmospheric oxygen levels (20%), as it is similar to oxygen levels in the adult brain [29]. GTICs were used at low passage numbers (less than 20 passages), as this has been shown to be important for maintaining multipotency [31]. Analysis of GTIC cultures from three different patients showed that they all expressed Lgl1 and PKCι, with expression of Lgl1 being considerably higher than in U87MG cells (Figure 4A). Cells from two patients showed detectable expression of PTEN, while cells from one patient (PriGO8A cells) showed a complete absence of PTEN expression, as in U87MG cells (Figure 4A). The latter were chosen for further detailed analysis. Chromogenic in situ hybridization showed that PriGO8A cells had three copies of the *EGFR* gene, likely reflecting a gain of chromosome 7, a characteristic genetic feature of glioblastoma (Figure 4B). When grown in the absence of laminin, the cells readily formed neurospheres resembling those seen in neural stem cell culture (Figure 4C). The cells also uniformly stained positive for nestin, a standard marker of neural stem cells (Figure 4D). When injected intracerebrally into immunocompromised mice, these cells formed a diffuse glioblastoma that was highly invasive (Figure 5A). The pattern of invasion was typical of glioblastoma, with extensive movement of cells into the uninjected hemisphere occurring along the corpus callosum. Thus these cells have the characteristic features of GTICs described in previous publications [5,32].

The ability of PriGO8A cells to differentiate in response to standard differentiation induction methods (serum addition, with or without growth factor withdrawal) was assessed (Figure 5B and C). To assess neuronal differentiation, neuron-specific class III β-tubulin (TUJ1) antibody was used; differentiation along the astrocytic lineage was assessed using antibody to glial fibrillary acidic protein (GFAP). These markers have been used extensively to assess differentiation along neuronal and astrocytic lineages in both GTICs and normal adult neural stem cells [3]. The addition of serum, either in the presence or absence of growth factors, increased the percentage of cells expressing TUJ1 and the percentage of cells expressing GFAP, indicating that PriGO8A cells differentiate along both neuronal and astrocytic lineages in the presence of serum. Although double labeling experiments were not performed here, the percentages of positive cells suggest that some of the differentiation observed is aberrant, giving rise to TUJ1/GFAP double positive cells, as observed previously [33,32].

### Effects of PTEN, PKCι and Lgl1 on GTIC differentiation

**Figure 6:**
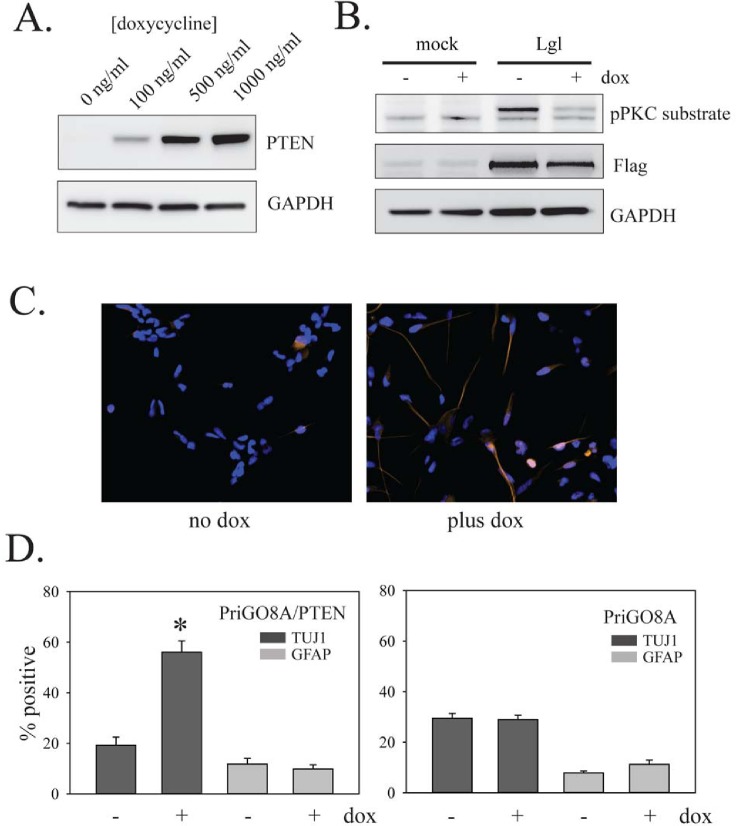
Effects of PTEN on PriGO8A differentiation A. PriGO8A cells were transduced with Tet activator lentivirus and selected with G418 for four days. Selected cells were then transduced with inducible PTEN lentivirus and selected with puromycin for four days. Selected cells were treated with the indicated concentrations of doxycycline for 48 h. Total cell lysates were then analyzed by Western blotting for expression of PTEN. B. PriGO8A cells with inducible PTEN were treated with 500 ng/ml doxycycline for two days and then either mock transduced or transduced with lentivirus constitutively expressing wild-type Lgl1. One day later cells were switched to media without EGF. Total cell extracts were collected one day later and analyzed by Western blotting with antibodies to phospho-PKC substrate and Flag epitope. C. PriGO8A cells with inducible PTEN were untreated or treated with 500 ng/ml doxycycline for 7 days. Cells were then fixed and immunocytochemistry for TUJ1 and GFAP expression was performed. D. Quantitation of data from C. The left graph shows doxycycline treatment of PriGO8A cells with inducible PTEN. The right graph shows doxycycline treatment of parental PriGO8A cells (as a control for effects of doxycycline alone). Quantitation was performed as described in Material and Methods. Data are shown as the mean ± SE. * indicates a p value less than 0.05.

To assess the effects of PTEN on differentiation, PriGO8A cells were engineered for inducible expression of PTEN as described above for U87MG cells. Treatment with doxycycline induced PTEN expression in a dose-dependent fashion (Figure 6A). As in U87MG cells, PTEN induction reduced the phosphorylation of transduced Lgl1 (Figure 6B). PTEN-induction in PriGO8A cells significantly increased the relative proportion of cells that were TUJ1 positive (Figure 6C and D). This was not seen in unmodified PriGO8A cells treated with doxycycline, showing that the effect is dependent on PTEN induction (Figure 6D). PTEN induction had no effect on differentiation along the astrocytic lineage (Figure 6D).

**Figure 7:**
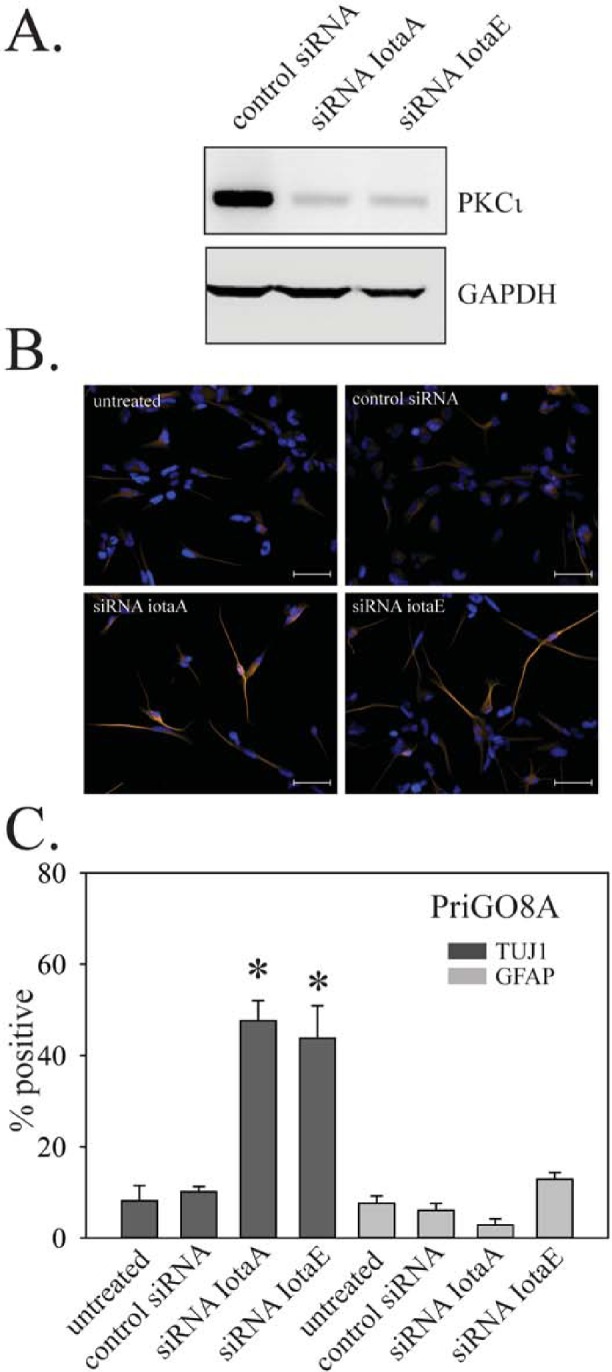
Effects of PKCι on PriGO8A differentiation A. PriGO8A cells were transfected with control RNA duplex or two different RNA duplexes targeting PKCι. Three days later, total cell lysates were collected and analyzed for PKCι expression by Western blotting. B. Cells were transfected as in A. Seven days later cells were fixed and immunofluorescence with antibodies to TUJ1 and GFAP was performed. Representative images of TUJ1 immunofluorescence are shown. C. Quantitation of TUJ1 and GFAP immunofluorescence seven days after PKCι knockdown. Quantitation was performed as described in Material and Methods. Data are shown as the mean ± SE. * indicates a p value less than 0.05.

To assess the role of PKCι in PriGO8A differentiation, transient knockdown of PKCι was performed using two different RNA duplexes at a 10 nM concentration (Figure 7A). Knockdown with both duplexes, but not with a control duplex used at the same concentration, resulted in increased differentiation along the neuronal lineage without any effects on astrocytic differentiation (Figure 7B and C).

**Figure 8:**
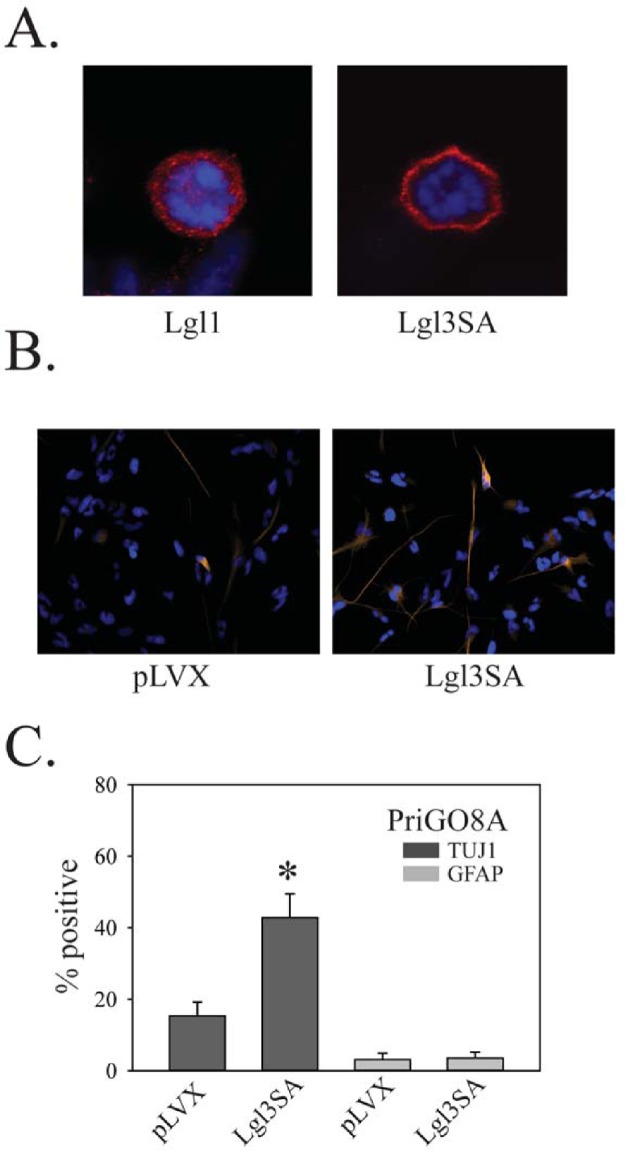
Effects of Lgl3SA on PriGO8A differentiation A. PriGO8A cells were transduced with either Lgl1 (left) or Lgl3SA (right). Four days later cells were fixed and immunofluorescence with anti-Flag antibody was performed. Representative examples of mitotic cells are shown. B. PriGO8A cells were transduced with either empty vector control (pLVX) or vector expressing Lgl3SA. Seven days later cells were fixed and immunofluorescence for TUJ1 and GFAP was performed. Representative images for TUJ1 staining are shown. C. Quantitation of TUJ1 and GFAP immunofluorescence seven days after transduction with Lgl3SA. Data are shown as the mean ± SE. * indicates a p value less than 0.05.

To assess the role of Lgl1 phosphorylation, PriGO8A cells were transduced with lentiviral vector expressing Lgl3SA. As with U87MG cells, Lgl3SA, but not Lgl1, showed marked membrane localization in mitotic cells (Figure 8A). As with PTEN transduction and PKCι knockdown, transduction of Lgl3SA also induced PriGO8A cell differentiation along the neuronal lineage, but not the astrocytic lineage (Figure 8B and 8C).

**Figure 9:**
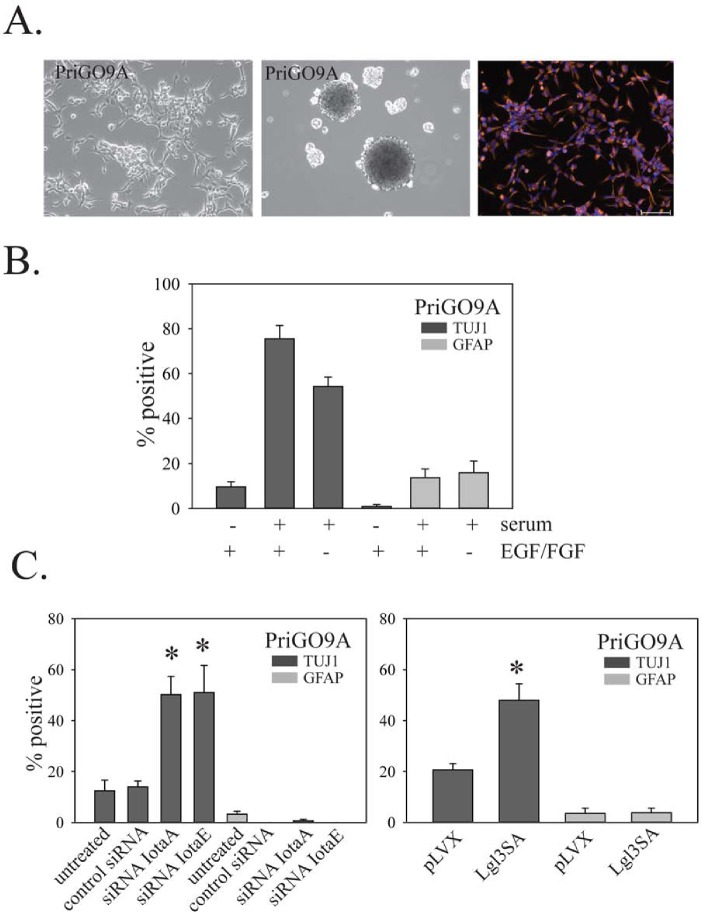
Effects of PKC and Lgl3SA on PriGO9A cell differentiation A. Morphology under phase contrast microscopy (top left panel), neurosphere formation (top middle panel) and nestin immunofluorescence (top right panel) for PriGO9A cell cultures. B. Differentiation of PriGO9A cells after exposure to serum with or without growth factor withdrawal, determined as described in Figure 5. C. Bar graphs show the effects of PKCι knockdown (left panel) and Lgl3SA transduction (right panel) on PriGO9A differentiation, determined as described in Figure 7.

**Figure 10:**
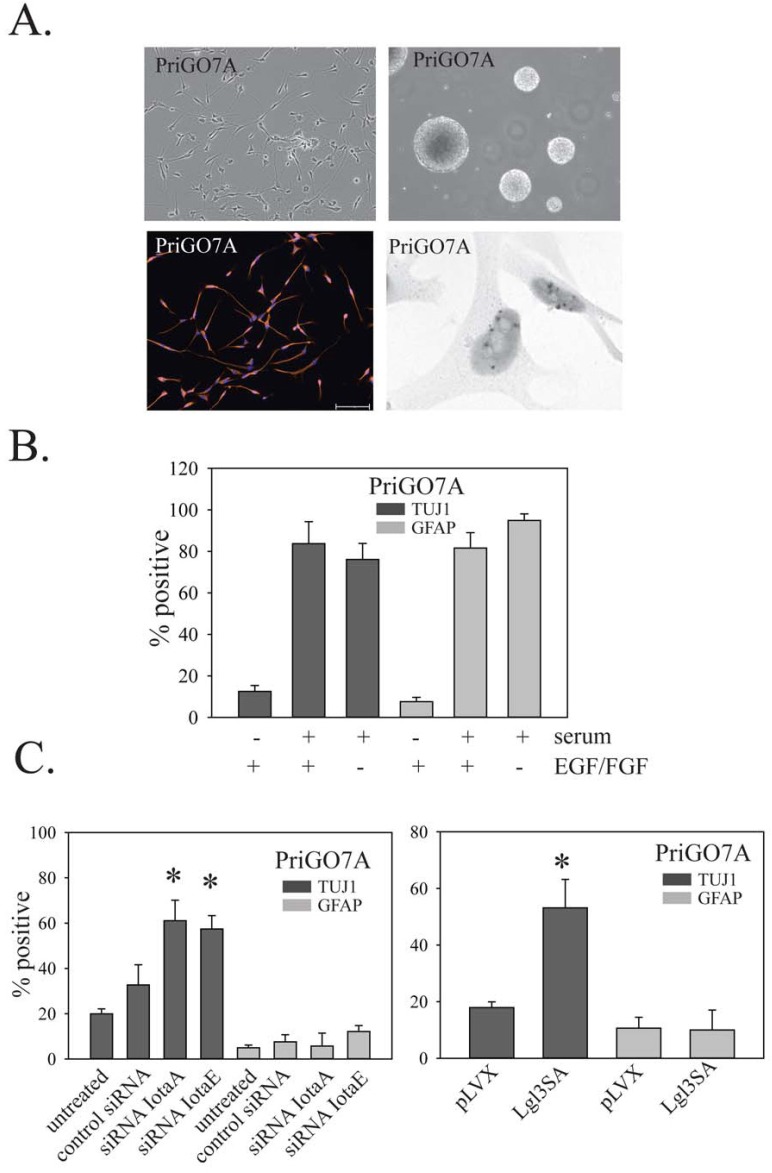
Effects of PKC and Lgl1 on PriGO7A differentiation Morphology under phase contrast microscopy (top left panel), neurosphere formation (top right panel), nestin immunofluorescence (lower left panel) and *EGFR* chromogenic in situ hybridization for PriGO7A cells (lower right panel). B. Differentiation of PriGO7A cells after exposure to serum or growth factor withdrawal. C. Bar graphs show the effects of PKCι knockdown (left panel) and Lgl3SA transduction (right panel) on PriGO7A differentiation.

GTICs from different patients have been reported to show variability in their differentiation behavior [5]. To determine the generalizability of the above findings, the effects of PKCι knockdown and Lgl3SA expression on GTIC differentiation were also assessed in cell populations isolated from two other patients, designated PriGO9A and PriGO7A. As with PriGO8A cells, PriGO9A and PriGO7A cells were able to form neurospheres when grown in the absence of laminin, were uniformly nestin positive and were able to undergo differentiation along neuronal and astrocytic lineages when exposed to serum (Figure 9A-B and 10A-B). Western blot analysis of PriGO9A and PriGO7A cells for expression of PTEN, Lgl1 and PKCι was shown in Figure 4A. Both show similar levels of PKCι and Lgl1 to those seen in PriGO8A cells. PriGO9A cells showed very low but detectable levels of PTEN, while PriGO7A cells show higher levels. PriGO7A cells were also examined by chromogenic in situ hybridization for *EGFR* copy number, which showed amplification in this region (Figure 10A). Both knockdown of PKCι and Lgl3SA transduction induced differentiation along the neuronal lineage in PriGO9A and PriGO7A cells without affecting astrocytic differentiation, similar to what was observed in PriGO8A cells (Figures 9C and 10C).

## DISCUSSION

Partial or complete loss of the tumor suppressor PTEN, by either hemizygous deletion, homozygous deletion or mutation, is a frequent event in glioblastoma. In this study, we demonstrate that loss of this tumor suppressor leads to the inactivation of a second protein with tumor suppressor-like functions, Lgl1. Previous work has shown both that Lgl is inactivated by phosphorylation and that the atypical PKCs are responsible for this [20,22]. The key finding here is the link between this and the loss of PTEN: we show that restoration of PTEN results in a decrease in Lgl1 phosphorylation as well as an increase in its membrane association, which is a marker of functional Lgl1. The effects of PTEN on Lgl1 phosphorylation were seen both in U87MG cells and in GTICs. This finding represents a second mechanism by which Lgl1 can be inactivated in cancer cells, as previous studies have shown that Lgl1 is downregulated at both the mRNA and protein levels in several cancer types, including colorectal cancer and melanoma, compared to normal tissue [24,25]. Lgl1 inactivation therefore appears to be a very frequent event in human cancers.

The effects of the PTEN/aPKC/Lgl1 pathway on the differentiation state of glioblastoma tumor initiating cells (GTICs) were assessed. GTICs were isolated from multiple patients using previously described culture techniques that preserve their ability to generate tumors in mice that mimic the pathology of human glioblastoma [32,5]. Cultures were screened for PTEN expression levels. GTICs from one patient (PriGO8A cells) were chosen for detailed study, as these showed complete loss of PTEN expression, in common with U87MG cells. PriGO8A cells showed the common features of GTICs that have been described previously in the literature [33,5], including nestin expression, the ability to form neurospheres, the ability to differentiate along both neuronal and astrocytic lineages in response to serum exposure, and the ability to form invasive tumors in immunocompromised mice.

PriGO8A cells were engineered for inducible expression of PTEN. As in U87MG cells, induction of PTEN in PriGO8A cells reduced the phosphorylation of Lgl1. Induction of PTEN also resulted in differentiation. This finding is similar to previous findings in adult neural stem cells and other non-cancer stem cell types, where loss of PTEN represses differentiation [34]. Although serum exposure resulted in differentiation of these cells along both neuronal and astrocytic lineages, with PTEN induction differentiation was exclusively along the neuronal lineage. This contrasts with the previously reported effects of BMP-4 on GTIC differentiation, where differentiation occurred primarily along the astrocytic lineage [35]. In most human glioblastoma tumors, the bulk of the cells are GFAP-positive, indicating that differentiation occurs primarily along the astrocytic lineage. Results here suggest the possibility that this is a consequence of strong repression of neuronal differentiation by PTEN loss and subsequent PI 3-kinase pathway activation.

As with PTEN induction, knockdown of PKCι also reduced the phosphorylation of Lgl1. In addition, knockdown of PKCι in PriGO8A cells resulted in differentiation along the neuronal lineage, phenocopying what was seen with PTEN induction. This indicates that PKCι is the key mediator of PI 3-kinase pathway effects on GTIC differentiation.

A non-phosphorylatable constitutively-active mutant of Lgl1 (designated Lgl3SA) was also expressed in PriGO8A cells. This was able to associate with the cell membrane; importantly this indicates that the human homologs of the *Drosophila* tumor suppressors Scribble and Discs large are functional in these cells, as these proteins are known to be required for Lgl membrane association [16]. As with PTEN induction and PKCι knockdown, expression of Lgl3SA induced differentiation exclusively along the neuronal lineage, indicating that Lgl1 is a key substrate mediating the effects of PKCι on GTIC differentiation.

The effects of Lgl3SA were also assessed in GTIC cells from two additional patients. PriGO9A cells, which showed low but detectable levels of PTEN, also underwent differentiation along a neuronal lineage when transduced with Lgl3SA. This effect was also seen in PriGO7A cells. These cells express high levels of PTEN, but show evidence of amplification of EGFR. Thus the role for Lgl1 does not appear to be restricted to GTICs in which PTEN expression is completely lost, but is also functional in GTICs where the PI 3-kinase pathway is hyperactivated by other mechanisms such as *EGFR* amplification.

The above findings for PKCι and Lgl1 are consistent with previous studies in *Drosophila* showing that atypical PKC and Lgl mediate the decision between neuroblast self-renewal and differentiation [17]. The data here show that this pathway is conserved in human GTICs and, critically, is linked to PTEN and PI 3-kinase signaling pathway status. The fact that partial or complete loss of PTEN expression is very common suggests that it is an early event in gliomagenesis. Thus inactivation of Lgl1 may also be an early event in gliomagenesis, leading to the expansion of a population of undifferentiated tumor initiating cells, the cell population that is the key driver for glioblastoma malignancy [2].

## MATERIALS AND METHODS

### Antibodies and chemicals

Anti-Flag M2 mouse monoclonal antibody, non-muscle myosin II rabbit polyclonal antibody and GFAP mouse monoclonal were from Sigma-Aldrich (Oakville, ON, Canada). PKCι mouse monoclonal and phospho-threonine 555 PKCι rabbit polyclonal antibodies were from BD Transduction Laboratories (Mississauga, ON, Canada) and Invitrogen (Carlsbad, CA, USA) respectively. The Akt goat polyclonal was from Santa Cruz Biotechnology (Santa Cruz, CA, USA). Phospho-AKT (Ser473) rabbit polyclonal, phospho-(Ser) PKC Substrate (P-S3-101) antibody (a mix of three rabbit monoclonal antibodies), and PTEN rabbit monoclonal antibody were from Cell Signaling Technology (Danvers, MA, USA). Lgl1 rabbit polyclonal antibody and GAPDH mouse monoclonal (6C5) antibody were from Abcam (Cambridge, MA, USA). TUJ1 rabbit monoclonal antibody was from Covance (Princeton, NJ, USA). Nestin mouse monoclonal antibody was from R&D Systems (Minneapolis, MN, USA).

### Cell Culture

U87MG cells were grown in Dulbecco's Modified Eagle medium supplemented with 100 units/ml penicillin, 100 μg/ml streptomycin and 10% fetal bovine serum at 37°C and 5% CO_2_. Cells were used at low passage number and were routinely checked and shown to be free of mycoplasma. GTIC cultures were isolated following a protocol approved by the Ottawa Hospital Research Ethics Board. Surgical samples were harvested from consented patients undergoing surgery for suspected glioblastoma (and without a history of previous lower grade brain tumor) using a Nico Myriad surgical device (NICO Corporation, Indianapolis, IN, USA). Cultures were digested with Accutase, filtered through 100 μM and 40 μM nylon mesh filters, and plated on laminin-coated plates as described by Pollard *et al*. [5]. Accutase and laminin were from Sigma-Aldrich, Oakville, ON, Canada. Cultures were grown in Neurobasal A medium supplemented with B27, N2 (all from Life Technologies, Burlington, ON, Canada), EGF and FGF (Peprotech, Rocky Hill, NJ, USA) at 37°C in 5% O_2_/CO_2_.

### Plasmid constructs

Full-length cDNA encoding Lgl1 mRNA was produced as described previously [14]. Site-directed mutagenesis was used to add an amino terminal Flag tag using the QuikChange XL Site-Directed Mutagenesis kit (Stratagene, La Jolla, CA, USA). Site-directed mutagenesis was also used to change the codons for serine 656, 660 and 664 to alanine to generate Flag-tagged Lgl3SA cDNA. For constitutive lentiviral vector production, Flag-tagged wild-type Lgl or Lgl3SA were PCR amplified to add Xba1 and Sal1 5' and 3' restriction sites and subcloned into pLenti-CMV GFP Puro (Addgene Plasmid 17448) which had been digested with the same restriction enzymes to remove the GFP cDNA. For inducible expression, PTEN cDNA, Flag-tagged Lgl cDNA and Flag-tagged Lgl3SA cDNA were subcloned into the Tet-inducible lentiviral vector pLVX-Tight-Puro (Clontech, Mountain View, CA, USA).

### Transduction with lentiviral vectors

Replication-incompetent lentiviral particles were made by the four-plasmid transfection method described by Wiederschain *et al*. [36]. U87MG were transduced by incubation for 24 h with lentivirus-containing supernatant added to regular media supplemented with 10 μg/ml polybrene (for U87MG transductions; polybrene was omitted for tumor initiating cell transductions). For inducible expression, cells were first transduced with lentivirus made with Tet-activator plasmid (Clontech, Mountain View, CA, USA) and selected with G418 (500μg/ml); these cells were then transduced again with lentivirus made with the inducible cDNA vectors described above and selected with puromycin (1μg/ml for U87MG and 0.5μg/ml for PriGO cells).

### Western blotting

Western blotting was done as described previously [15]. Chemiluminescence from HRP conjugated secondary antibodies was detected with the Alpha Innotech Fluorchem system (Santa Clara, CA, USA) and quantitated using Alphaview software.

### Immunofluorescence microscopy

Immunofluorescence microscopy was done as described previously [14]. For differentiation experiments using TUJ1 and GFAP antibodies, images were taken of five random fields of view (chosen under DAPI filter) per condition per experiment. Analysis was carried out in ImageJ software (National Institutes of Health, Bethesda, Maryland, USA). The proportion of positive cells to total nuclei was assessed per condition +/− SE for each experiment.

### Chromogenic in situ hybridization

Chromogenic in situ hybridization was used to detect EGFR gene copy number changes in PRIGO cells. ZytoDot SPEC *EGFR* Probe (Cat. ZTV-C-3007-400) and ZytoDot CISH Implementation Kit were from Cedarlane (Burlington, ON, Canada) and were used according to the manufacturer's recommendations.

### Intracerebral xenografts

Experiments were carried out in accordance with the recommendations of the Animal Care Committee at the University of Ottawa.

5 × 10^5^ PriGO cells in 10μL sterile PBS were injected intrastriatal (right side of the skull approximately 0.5mm above the coronal suture and 2mm from the sagittal suture) into 4-6 week old SCID/beige mice (Charles River Laboratories, Wilmington. MA). Endpoint was reached after approximately three months when mice became symptomatic. Whole brains were harvested, formalin fixed and paraffin embedded. Sections were cut, stained with hematoxylin and eosin, and reviewed by a neuropathologist (JW).

### Statistics

SigmaPlot12 software was used for statistical analyses. Comparisons between two groups were performed using two-tailed t-tests with a p value <0.05 considered significant.
